# The Association of Neighborhood-Level Deprivation with Glioblastoma Outcomes: A Single Center Cohort Study

**DOI:** 10.21203/rs.3.rs-5913656/v1

**Published:** 2025-01-30

**Authors:** Yifei Sun, Dagoberto Estevez-Ordonez, Travis J Atchley, Burt Nabors, James Markert

**Affiliations:** University of Alabama at Birmingham; University of Alabama at Birmingham; University of Alabama at Birmingham; University of Alabama at Birmingham; University of Alabama at Birmingham

**Keywords:** Area deprivation, Glioblastoma, neighborhood socioeconomic status, survival

## Abstract

**Purpose:**

Glioblastoma is the most common primary brain malignancy. Though literature has suggested the association of glioblastoma outcomes and socioeconomic status, there is limited evidence regarding the association of neighborhood-level socioeconomic deprivation on glioblastoma outcomes. The aim of this study was to assess the impact of neighborhood-level socioeconomic deprivation on glioblastoma survival.

**Methods:**

We retrospectively reviewed all adult glioblastoma patients seen at a single institution from 2008 to 2023. Neighborhood deprivation was assessed via Area Deprivation Index (ADI), with higher ADI indicating greater neighborhood socioeconomic deprivation. Log-rank tests and multivariate cox regression was used to assess the effect of ADI and other socioeconomic variables while controlling for *a priori* selected clinical variables with known relevance to survival.

**Results:**

In total, 1464 patients met inclusion criteria. The average age at diagnosis was 60 ± 14 years with a median overall survival of 13.8 months (IQR 13–14.8). The median ADI of the cohort was 66(IQR 46–84). Patients with high ADI had worse overall survival compared to patients with low ADI (11.7 vs 14.8 months, p=.001). In the multivariable model, patients with high ADI had worse overall survival (HR 1.25, 95%CI 1.09–1.43). To account for changes in WHO guidelines, we implemented the model on patients diagnosed between 2017–2023 and findings were consistent (HR 1.26,95%CI 1.01–1.56).

**Conclusion:**

We report the first study demonstrating glioblastoma patients with higher neighborhood deprivation have worse survival after controlling for other socioeconomic and biomolecular markers. Neighborhood socioeconomic status may be a prognostic marker for glioblastoma survival.

## INTRODUCTION

Glioblastoma is the most common primary brain malignancy, comprising around half of all primary brain tumors [1]. Despite recent progress in treatments, prognosis for patients remain poor, with median survival around 15 months [2]. Thus, it is of interest to better understand the risk factors that are associated with worsened survival. Recent literature has identified a connection between socioeconomic disparity and worsened outcomes in patients with glioblastoma [3, 4].

Socioeconomic factors are complex and can differentially affect outcomes. Recent studies have identified racial and income-related disparities in surgical care, chemotherapy, and radiotherapy for patients in glioblastoma [4, 5]. Other studies have also identified disparities in glioblastoma outcomes on the basis of race, insurance status, age, and educational status, as well as inequalities in timeliness of care as well [6].

However, the effect of neighborhood-level SES status on glioblastoma survival remains unclear. Much of socioeconomic literature data at the ZIP code level, which has been shown to be poor proxies of socioeconomic status [7]. Furthermore, currently commonly utilized measures of SES are often poorly generalizable and subject to regional bias [8, 9]. Area Deprivation Index (ADI) is a tool developed by the Health Resources and Services Administration (HRSA) that measures neighborhood-level disadvantage by taking into account 17 measures of socioeconomic disparity in 4 main domains: education, income/employment, housing, and household characteristics [10].

Amongst the most studied area-level measures of socioeconomic disadvantage and independently validated by studies across many domains of health outcomes research, neighborhood deprivation has been used to link socioeconomic disparity to poor patient outcomes in diabetes research, cardiovascular research, and other surgical specialties [11–13]. Neighborhood deprivation has also gained increased attention due its inclusion in incentives programs and reimbursement adjustment calculations by the Center for Medicare/Medicaid (CMS) [14].

Despite its widespread adoption in other fields, there is little evidence regarding the association of neighborhood-level socioeconomic status on the overall survival (OS) of patients with glioblastoma. There is also little literature that examine the effect of these socioeconomic factors on glioblastoma outcomes in the context of clinically important molecular markers such as MGMT methylation and IDH wild-type status.

The aim of this study was to assess the impact of neighborhood-level socioeconomic status on glioblastoma survival in the largest cohort to date and to better understand how socioeconomic status affects outcomes for patients with glioblastoma. To our knowledge, this is the first report to describe the association of neighborhood level socioeconomic deprivation with OS in glioblastoma.

## METHODS

We performed a single center retrospective review with approval from the Institutional Review Board (IRB- 300011516). This manuscript was written in compliance with STROBE (Strengthening the Reporting of Observational Studies in Epidemiology) [15].

### Participants and Data Collection

We retrospectively identified all adult patients, 18 years or older, with histopathological new glioblastoma diagnosis seen at our institution between January 1^st^, 2008 and December 31^st^, 2023. In total, 1493 patients met inclusion criteria. The electronic medical record (EMR) was reviewed for variables on patient demographics, socioeconomic background, geography, and treatment characteristics. Due to the retrospective nature of this study, patient consent was not needed.

### Defining Variables

Variables were defined *a priori* with advice from the senior authors. Study variables included were age at diagnosis, race, gender, marital status, extent of surgical resection, IDH status MGMT methylation status, history of chemotherapy and history of radiotherapy. Patient addresses were extracted from the EMR and were geocoded using ArcGIS software. Federal Information Processing System (FIPS) codes were extracted and correlated to its individual Area Deprivation Index (ADI), with higher ADI relating to more socioeconomic deprivation. ADI was retrieved from the Neighborhood Atlas dataset produced by the Center for Health Disparities Research at the University of Wisconsin School of Medicine and Public Health.^4^ High ADI was designated patients in the top national quartile according to previous literature [16].

### Statistical Analysis

Univariable analysis including Student’s t-test, one-way analysis of variance (ANOVA), Chi squared test, and Wilcoxon rank sum test were used to compare incidences of chemotherapy, radiotherapy extent of resection, and other demographic variables between patients with high and low ADI. Kaplan Meier curves were used to investigate differences in survival between groups of interest, and log rank tests were used to assess differences in survival.

**Figure 1:**
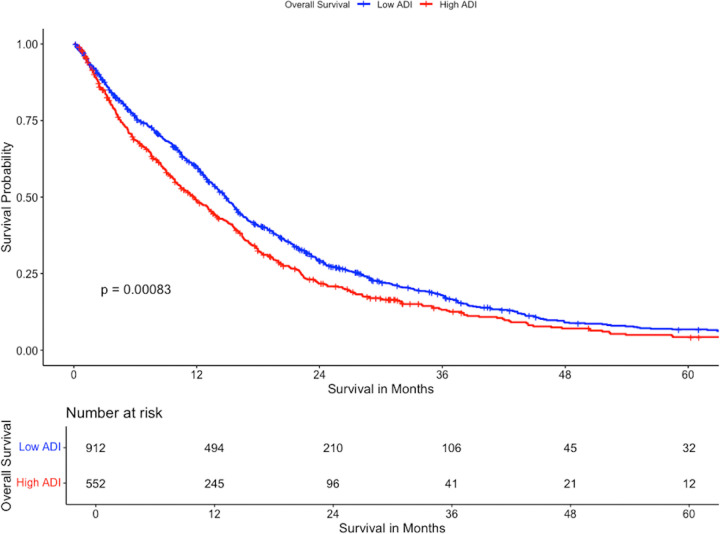
Kaplan Meier Survival Plot stratified by ADI

**Figure 2:**
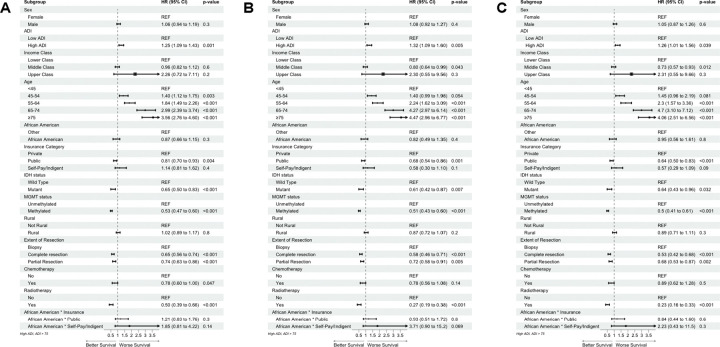
Forest Plot of Multivariate Cox Regression Analysis for Overall Survival **A.**Imputed cohort **B.** Complete Case cohort **C.** Post-2016 WHO guidelines cohort

Sensitivity analysis was conducted by performing multiple methods of imputation for missing data as well as replicating the model in patients diagnosed and treated after the WHO guidelines in 2016. All statistical analyses were performed using R studio (version 4.3.1) [17]. Further details on statistical analysis can be found in the supplementary content (Supplementary Content, Table 1S – 5S, Figure 1S–2S).

## RESULTS

In total, 1464 patients met inclusion criteria. The mean age at diagnosis was 60 ± 14 years. Of these patients, 155 (11%) were African American (AA) and 816 (56%) were male. At time of censoring, 249 (17%) were alive. Of these patients, 671(46%) received complete resection, 1235 (84%) received radiotherapy and 1219 (83%) received chemotherapy. The median ADI was 66 (IQR 46–84). Ninety-two (6.3%) of the patients had IDH mutations and 344 (23%) of the patients were of MGMT-methylated status. The median OS (mOS) of the cohort was 13.7 months (IQR 12.99–14.16). Further details on patient demographics and characteristics can be found in the Table 1 and supplement (Supplementary Content, Table 6S).

### Univariable Comparison Analysis

Patients in the highest quartile of ADI were more likely to be AA (17% vs 6.6%, p<.001), higher rates of Medicaid/Medicare (52% vs 45.6%, p<.001), more likely to be MGMT unmethylated (33% vs 40%, p=.037), more likely to live greater than 60 miles from the institution, and more likely to live in a rural region (7.8% vs 0.9% p<.001). These patients were also less likely to undergo complete resection compared to those with lower ADI (42% vs 48%, p=.021). The results of this analysis can be found in Table 1.

### Univariable Survival Comparison Analysis

In univariable survival comparison, AA patients had longer mOS (15.2 months, 95%CI 10.6–18.0) compared to Caucasian patients (13.5 months 95%CI 12.6 – 14.5). Patients with high ADI had lower mOS (11.67 months, 95% CI 13.8–15.8) compared to those with low ADI (14.83 months, 95%CI 10.2–13.4). Patients who were privately insured had longer mOS (15.4 months, 95%CI 14.5 – 16.5) compared to those who were publicly insured (11.5 months, 95%CI 10.0–12.9) and those who were uninsured (12.4 months, 95%CI 7.3–21.2). Patients who received chemotherapy had longer mOS (15.4 months, 95%CI 14.6 – 16.1) than those who did not (3.7 months, 95%CI 3.1–4.7). Patients who received radiotherapy had longer mOS (15.6 months, 95%CI 14.5–16.5) compared to those who did not (3.2 months, 95%CI 3.0–4.0). The results of this analysis can be found in Table 2. Kaplan Meier survival curves stratifying for ADI are presented in Figure 1.

### Multivariate cox proportional hazards

Using multivariate cox proportional hazards analysis, we identified that patients with high ADI had worse survival (HR 1.26, 95%CI 1.11–1.43, p<.001) compared to patients with low ADI. These findings were replicated in both types of imputation, complete case analysis, and in subgroup analysis of patients diagnosed after the 2016 WHO CNS Guidelines. (Supplementary Content, Table 7S) When compared to patients who were in the youngest age group at time of diagnosis (<45 years), all other age groups had worse survival. Patients who underwent gross total resection had better survival (HR 0.65, 95%CI 0.56–0.74, p <.001) compared to patients who underwent partial resections or biopsy. Patients with public insurance had better survival (HR 0.81, 95%CI 0.70–0.93, p=.004) compared to patients with private insurance. Patients with IDH mutation had better survival (HR 0.65, 95%CI 0.56–0.74, p<.001) compared to those with wild-type IDH. Patients with methylated MGMT had better survival (HR 0.53, 95%CI 0.47–0.61, p<.001) when compared to patients with unmethylated MGMT. In all three multivariate models, AA race was not found to be associated with worsened survival. Chemotherapy was found to be associated with improved survival in only the MICE model (HR 0.74, 95%CI 0.57–0.95, p=.02). Forest plots for this analysis can be found in Figure 2.

## DISCUSSION

Here we report the first analysis of the effects of neighborhood level socioeconomic status on glioblastoma survival after adjusting for other socioeconomic, clinical, and molecular factors. Our findings strongly suggest that neighborhood deprivation independently predicts survival in patients with glioblastoma and is a potential prognostic marker for patients with glioblastoma.

The role of socioeconomic status in cancer is well known, with many studies highlighting the effect of socioeconomic status on survival of cancer patients. Studies have suggested that income, insurance status, and various other commonly used measures are imperfect proxies of socioeconomic status, varying by region and race. Studies on the association of race and other socioeconomic markers are conflicted as well. Commonly utilized measures of socioeconomic disadvantage in literature can be imperfect proxies for SES [8, 9]. Additionally, social determinants of health play complex roles in determining the health outcomes of patients, and findings may be difficult to generalize findings on a national level. Thus, the utilization of ADI represents an advancement in understanding the prognostic effect of patient SES on glioblastoma survival by utilizing a nationally standardized, multifactorial measure of patient socioeconomic disadvantage.

Here, we report the effect of ADI, an accurate and well-validated measure of neighborhood-level socioeconomic status, with survival in patients with glioblastoma in the largest single institution cohort to date. ADI has emerged as a preferred metric for capturing socioeconomic status in many fields of medicine [12, 18, 19]. However, it has been under-utilized in neuro-oncology and neurosurgery. We observed that high ADI is independently associated with worse survival compared to patients with lower ADI (HR 1.25, 95% CI 1.09– 1.43, p<.001) after adjusting for age, race, income, insurance status, IDH status, MGMT methylation, rurality, extent of resection, history of chemotherapy, and history of radiotherapy. There are many potential mechanisms for this observation.

### Access to Care

Patients with high ADI reflect high degrees of socioeconomic disparity, which has been associated with decreased neurosurgical coverage [20]. This is reinforced in our findings, in which we observed that patients with high ADI were less likely to have undergone gross total resection compared to patients with high ADI (Table 2). Analysis by Perla et al.[21] found that patients with higher ADI had lower access to post-operative care in glioblastoma patients, reflecting a disparity in access to neurosurgical care. Similarly, a study conducted by Guidry et al.[22] found that patients with high ADI patients were more likely to be lost to follow-up and have an unplanned readmission following emergent surgery for acute subdural hematoma. Barriers in accessing to care would contribute to a worse prognosis for glioblastoma patients, whether due to socioeconomic or geographic disparities. The association of transportation difficulties would potentially be associated with poor glioblastoma outcomes as well, though we adjusted for rurality, and by proxy, distance in our analysis.

### Adherence to Treatment

High ADI is associated with difficulties in adhering to treatment regimens. An analysis conducted by Brown et al. [23] found that patients with high ADI had increased rates of no-shows in scheduled telehealth visits. A study by Hensley et al.[24] suggested that patients of low socioeconomic status had lower rates of medication adherence. Similar results were found by Wadhwania et al.[25] in children following liver transplantation, where patients with higher neighborhood-level socioeconomic deprivation had lower levels of medication adherence. Neighborhood-level socioeconomic deprivation may capture barriers in patient education, access to care, or a socioeconomic environment that may present difficulties in adhering to treatment regiments [26]. This association in the context of glioblastoma treatment should be further explored in future studies.

### Clinical Comorbidities

High ADI has also been shown to be associated with comorbidities such as uncontrolled diabetes and cardiovascular health. Durfey et al. [27] reported that patients with high ADI had more and worse controlled chronic comorbidities. Similarly, Kurani et al. [28] identified that diabetes patients with high ADI received lower-quality care, leading lower significantly lower likelihood of acceptable HbA1C levels, blood pressure, and lipid levels. Additionally, they identified that increased ADI was also associated with smoking. Lindner et al.[29] also found that ADI was associated with lower glycemic control in patients with Type I Diabetes Mellites (DMI) and higher risk of diabetic ketoacidosis. This was also found by Rodriguez et al.[30], who identified an increased incidence of cardiovascular comorbidities in patients with high ADI. Worsened comorbidity status could result in worse tolerance of the challenging and taxing course of treatment that is the current standard of care for glioblastoma patients, leading to earlier mortality, which is supported in analysis by Carr et al.[31]

### Health Literacy

High ADI has also been associated with lower levels of health literacy as well. Knighton et al. [32] found that low health literacy was significantly associated with a higher ADI. The relationship between low health literacy and poor health outcomes is well-established in the literature. Poor health literacy may contribute to delays in presentation, lower rates of follow-up care, and lower rates of adherence to treatment regimens [33, 34]. However, the association of health literacy and glioblastoma survival should be investigated in future studies to better understand this mechanism.

### Delayed Care

High ADI could result in delayed initiation of care of glioblastoma, leading to delayed detection and a worse prognosis. Areas of high ADI generally have lower primary care coverage, and these socioeconomically disadvantaged patients are less likely to be insured and have regular primary care providers (PCPs); this would lead to delays in diagnosis and treatment of glioblastoma in patients with high ADI and potentially lead to worse outcomes [35]. The association of low socioeconomic and lack of oncological and neurosurgical care is also well established [20, 36, 37]. This is supported by analysis by Aguirre et al. [38] who reported that patients with high ADI had higher WHO tumor grades at presentation. Delay in cancer care for socioeconomically disadvantaged patients has also been identified in previous literature as well. In an analysis conducted by Ahmad et al. [39], patients of public insurance, and racially minoritized patients were more likely to encounter delays in initiation of treatment for anal squamous cell carcinoma.

### Interventions

Utilization of neighborhood-level deprivation offers several avenues for systematic interventions to ameliorate disparities in survival. ADI captures patients who may have lower levels of education, income and employment, issues with housing, and disparate housing conditions such as lack of access to internet or phones. Thus, interventions may be designed to address each aspect of neighborhood deprivation captured by ADI (Supplementary Content, Table 8S).

To address patients with low educational attainment and health literacy, increased outreach and public education programs should be put in place in communities with high neighborhood deprivation to allow for potentially earlier detection, improved decision-making, and better overall management of disease progression [40]. Assistance with access to primary care and preventative services, enrollment in public food assistance programs and other community programs have also been shown to be of use in improving health outcomes and may be an effective intervention for patients with high neighborhood deprivation [41, 42]. To address the patients with disadvantaged housing characteristics, implementation of medical-legal collaborations and assistance in accessing public housing unites may help address inequitable housing conditions and improve outcomes for patients as well [43, 44]. Assistance through low-cost/free internet access programs, as well as transportation assistance programs may help decrease rates of follow-up loss and improve survival in patients who struggle with low household characteristics [45]^,^[46–48].

Patients with high ADI may encounter delays of care for glioblastoma, contributing to a worse overall prognosis. Future studies should investigate the relationship between neighborhood socioeconomic status and time to presentation or degree of glioblastoma progression at initial evaluation to better understand this mechanism.

### Limitations

Our study is limited by its retrospective design. New revised 2021 WHO Central Nervous System (CNS) Tumor guidelines categorize IDH mutant, grade IV astrocytomas as a separate entity from glioblastoma. All IDH-mutant tumors were still included in this cohort to understand the socioeconomic disparities that exist in high grade glioma care. However, we controlled for IDH status in our analysis. There was missing data for IDH status and MGMT methylation status in our cohort, largely due to changing patterns of practice and the diagnosis and treatment of patients prior to the adoption of 2016 WHO CNS tumor guidelines. Because of this, we can reasonably suspected that missing data patterns likely met criteria for missing-at-random (MAR), thus justifying the usage of multiple imputations even at higher proportions [49, 50]. Furthermore, two different methods of imputation, complete case analysis, and a separate analysis using only patients diagnosed after the 2016 WHO CNS guidelines were consistent, reinforcing the robustness of our findings. Our study also does not consider quality of life and functional metrics, which may be associated with survival and may be considered as future avenues of study. Though ADI is a highly validated measure of socioeconomic status in medicine, it may fail to completely capture other aspects of a patient’s socioeconomic status. Despite larger numbers, single study design may suggest possible selection bias for patients seen at a high-volume academic center.

## CONCLUSION

In this study, we have validated ADI as a potential prognostic marker for overall survival in glioblastoma. Utilization of ADI allows more nuanced and granular understanding of socioeconomic status in patients with glioblastoma. Patients with high ADI should be considered at higher risk of poor outcomes and received additional counseling by an interdisciplinary team. Future studies should seek to further validate the effect of ADI on glioblastoma outcomes in a multi-center cohort and to identify interventions to remedy this disparity.

## Figures and Tables

**Table 1.** Patient Characteristics and Demographics

	High ADI *		
	No, N = 912^1^	Yes, N = 552^1^	p-value^2^
Age (years)			0.8
< 45	124 (14%)	87 (16%)	
45–54	142 (16%)	89 (16%)	
55–64	248 (27%)	144 (26%)	
65–74	281 (31%)	164 (30%)	
≥75	117 (13%)	68 (12%)	
Sex			0.5
Female	398 (44%)	250 (45%)	
Male	514 (56%)	302 (55%)	
Race			<0.001
Black	60 (6.6%)	95 (17%)	
Other	62 (6.8%)	23 (4.2%)	
White	790 (87%)	434 (79%)	
Married	682 (75%)	351 (64%)	<0.001
Insurance			<0.001
Indigent/Self Pay	28 (3.1%)	23 (4.2%)	
Medicaid	51 (5.6%)	60 (11%)	
Medicare	365 (40%)	225 (41%)	
Private	468 (51%)	244 (44%)	
Median Household Income (USD)	55,681 (47,276, 68,608)	42,116 (37,433, 48,371)	<0.001
Vital Status			0.12
Alive	166 (18%)	83 (15%)	
Deceased	746 (82%)	469 (85%)	
IDH status			0.4
IDH-Mut	55 (6.0%)	37 (6.7%)	
IDH-WT	566 (62%)	324 (59%)	
Unknown	291 (32%)	191 (35%)	
MGMT Status			0.037
Methylated	211 (23%)	133 (24%)	
Unknown	340 (37%)	236 (43%)	
Unmethylated	361 (40%)	183 (33%)	
Extent of Resection			0.021
Biopsy	247 (27%)	183 (33%)	
Complete resection	441 (48%)	230 (42%)	
Partial Resection	224 (25%)	139 (25%)	
Received Radiotherapy	782 (86%)	453 (82%)	0.06
Received Chemotherapy	770 (84%)	449 (81%)	0.12
ADI	52 (36, 64)	87 (82, 92)	<0.001
RUCA			<0.001
Metropolitan	753 (83%)	309 (56%)	
Micropolitan	111 (12%)	114 (21%)	
Rural	8 (0.9%)	43 (7.8%)	
Small Town	40 (4.4%)	86 (16%)	
Distance from Institution (miles)			<0.001
<60	426 (47%)	191 (35%)	
60–200	362 (40%)	336 (61%)	
≥200	124 (14%)	25 (4.5%)	

1 n (%); Median (IQR)

2 Pearson’s Chi-squared test; Wilcoxon rank sum test.

* ADI-Area Deprivation Index, High ADI-ADI>75

**Table 2.** Univariable Survival Analysis


	Level	Median survival	CI lower	CIupper	p-value^1^
Age	< 45	26.27	22.03	32.12	<.001
	45–54	17.69	15.85	20.51	
	55–64	14.66	13.08	16.18	
	65–74	9.86	8.38	11.28	
	≥75	6.12	4.67	7.53	
Sex	Female	14.10	12.53	15.48	0.25
	Male	13.51	12.79	14.83	
Income Class*	Lower	13.84	13.02	15.06	0.51
	Middle	13.51	11.24	15.09	
	Upper	10.32	1.12	NA	
ADI**	Low ADI	14.83	13.81	15.78	<.001
	High ADI	11.67	10.22	13.38	
IDH status	IDH-Mut	33.57	27.32	41.79	<.001
	IDH-WT	13.08	12.09	14.14	
	Unknown	13.12	11.80	14.89	
MGMT Status	Methylated	21.14	18.35	23.08	<.001
	Unmethylated	12.99	11.74	14.10	
	Unknown	12.26	10.49	13.48	
Extent of Resection	Biopsy	7.43	5.85	8.84	<.001
	Complete resection	17.03	16.14	19.04	
	Partial Resection	14.01	12.33	15.58	
Chemotherapy	No	3.68	3.12	4.67	<.001
	Yes	15.42	14.63	16.14	
Radiotherapy	No	3.22	3.02	4.04	<.001
	Yes	15.55	14.70	16.21	
Insurance Category	Private	15.39	14.53	16.50	<.001
	Public	11.51	9.99	12.85	
	Self-Pay/Indigent	12.36	7.27	21.17	
RUCA***	Metropolitan	14.24	13.12	15.16	0.18
	Micropolitan	13.71	11.74	15.75	
	Rural	11.57	9.04	19.96	
	Small Town	11.97	9.50	15.52	
Race	Black	15.19	10.62	17.98	0.13
	White	13.51	12.59	14.53	
	Other	18.94	13.58	23.80	

1 log-rank test;

* Income categories determined according to Pew Research Reports 2022;

** ADI- Area Deprivation Index;

*** RUCA-Rural Urban Communicating Area

## Data Availability

Data is available upon reasonable request.

## Abbreviations

ADI: Area Deprivation Index
ERS: Economic Research Service
FIPS: Federal Information Processing Standards
HRSA: Health Resources and Services Administration
